# Protection of Kidney Function with Human Antioxidation Protein α_1_-Microglobulin in a Mouse ^177^Lu-DOTATATE Radiation Therapy Model

**DOI:** 10.1089/ars.2018.7517

**Published:** 2019-03-29

**Authors:** Amanda Kristiansson, Jonas Ahlstedt, Bo Holmqvist, Anders Brinte, Thuy A. Tran, Eva Forssell-Aronsson, Sven-Erik Strand, Magnus Gram, Bo Åkerström

**Affiliations:** ^1^ Division of Infection Medicine, Department of Clinical Sciences in Lund, Lund University, Lund, Sweden.; ^2^ ImaGene-iT AB, Medicon Village, Lund, Sweden.; ^3^ Lund University Bioimaging Center, Lund, Sweden.; ^4^ Department of Clinical Neuroscience, Karolinska Institutet, Stockholm, Sweden.; ^5^ Department of Radiation Physics, Institute of Clinical Sciences, Sahlgrenska Cancer Center, University of Gothenburg, Sweden.; ^6^ Medical Radiation Physics, Department of Clinical Sciences in Lund, Lund University, Lund, Sweden.; ^7^ Pediatrics, Department of Clinical Sciences in Lund, Skane University Hospital, Lund University, Lund, Sweden.

**Keywords:** α_1_-microglobulin, PRRT, renal protection, ^177^Lu-DOTATATE, cancer, radionuclide therapy

## Abstract

***Aims:*** Peptide receptor radionuclide therapy (PRRT) is in clinical use today to treat metastatic neuroendocrine tumors. Infused, radiolabeled, somatostatin analog peptides target tumors that are killed by irradiation damage. The peptides, however, are also retained in kidneys due to glomerular filtration, and the administered doses must be limited to avoid kidney damage. The human radical scavenger and antioxidant, α_1_-microglobulin (A1M), has previously been shown to protect bystander tissue against irradiation damage and has pharmacokinetic and biodistribution properties similar to somatostatin analogs. In this study, we have investigated if A1M can be used as a renal protective agent in PRRT.

***Results:*** We describe nephroprotective effects of human recombinant A1M on the short- and long-term renal damage observed following lutetium 177 (^177^Lu)-DOTATATE (150 MBq) exposure in BALB/c mice. After 1, 4, and 8 days (short term), ^177^Lu-DOTATATE injections resulted in increased formation of DNA double-strand breaks in the renal cortex, upregulated expression of apoptosis and stress response-related genes, and proteinuria (albumin in urine), all of which were significantly suppressed by coadministration of A1M (7 mg/kg). After 6, 12, and 24 weeks (long term), ^177^Lu-DOTATATE injections resulted in increased animal death, kidney lesions, glomerular loss, upregulation of stress genes, proteinuria, and plasma markers of reduced kidney function, all of which were suppressed by coadministration of A1M.

***Innovation and Conclusion:*** This study demonstrates that A1M effectively inhibits radiation-induced renal damage. The findings suggest that A1M may be used as a radioprotector during clinical PRRT, potentially facilitating improved tumor control and enabling more patients to receive treatment.

## Introduction

The human protein α_1_-microglobulin (A1M) is a physiological radical scavenger and antioxidant (1a). Mainly synthesized in the liver, A1M is a 26-kDa glycoprotein found in all vertebrates. A free, unpaired thiol group provides A1M with a strong reduction potential (2) and radical trapping mechanism (1b). As a result, A1M functions as a physiological antioxidant and the protein has been shown to protect cells and tissues against oxidative damage (22). Of particular importance, *in vitro* cell studies have shown that A1M can protect cells against propagation of α-particle irradiation-induced damage by suppressing cell death, apoptosis, and oxidative stress (21).

Innovation Peptide receptor radionuclide therapy (PRRT) is used to treat metastatic tumors in patients. This generates oxidative stress and free radicals that damage healthy tissue, especially in kidneys where peptides are retained. In this study, we show that coadministrating ^177^Lu-DOTATATE with the human radical scavenger α1-microglobulin (A1M) ameliorates both DNA damage and kidney damage as well as increases overall survival by protecting against oxidative damage caused by irradiation. This suggests that coadministrating A1M could potentially improve clinical PRRT by allowing higher treatment activity and making it accessible for patients today excluded from therapy due to renal insufficiency.

Today, cancer patients with inoperable somatostatin receptor-expressing neuroendocrine tumors can be treated with peptide receptor radionuclide therapy (PRRT) (3). In this procedure, infused radiolabeled somatostatin analogs, for example, [^177^Lu-DOTA^0^,Tyr^3^]octreotate (lutetium 177 [^177^Lu]-DOTATATE), target tumors by binding to tumor-expressed somatostatin receptors, driving tumors into regression due to the resulting high absorbed dose. The small size of somatostatin peptides, however, also allows glomerular filtration and tubular reabsorption, resulting in high absorbed doses in kidneys (19). The kidneys, along with bone marrow, are therefore dose-limiting organs in PRRT and primarily dictate the amount of administered activity given during therapy. Due to high absorption in kidneys, patients with known risk factors such as poor renal function, hypertension, and diabetes are currently excluded from PRRT treatment (4).

Current guidelines, adopted from the external beam radiation therapy, set the limit of the absorbed dose in kidneys at 18 Gy (4, 32). A recently published study indicates that current dosimetry models overestimate the absorbed dose to kidneys during fractionated ^177^Lu-DOTATATE PRRT and that patients receiving higher doses show improved outcome (27).

We recently demonstrated that human recombinant A1M displays a similar biodistribution pattern and pharmacokinetic behavior to the somatostatin analog octreotide with a high uptake in kidneys, predominantly in the renal cortex (1). All these features suggest that A1M can be used as a radioprotector of kidneys to prevent radiation-induced nephrotoxic side effects during PRRT.

In this work, we hypothesize that A1M can act as a radioprotector of kidneys during PRRT by decreasing the initial cellular damage and thereby protecting against subsequent development of nephropathy. We therefore investigated the short-term (1–8 days) and long-term (6–24 weeks) renal responses following injection of 150 MBq ^177^Lu-DOTATATE, with or without coadministration of human recombinant A1M, in a mouse model. The injected dose of A1M was set to 7 mg/mL, designed to result in a *C*_max_ value in blood of around 10 μ*M* in mice (1c), which has been shown to yield near-maximal protection effects *in vitro* and *in vivo* (1c, 21, 22). The results suggest that *in vivo* coadministration of A1M is a therapeutically effective method of reducing kidney damage in a preclinical ^177^Lu-DOTATATE radiation therapy model.

## Results

### Survival statistics and postmortem observations

**FIG. 1.**
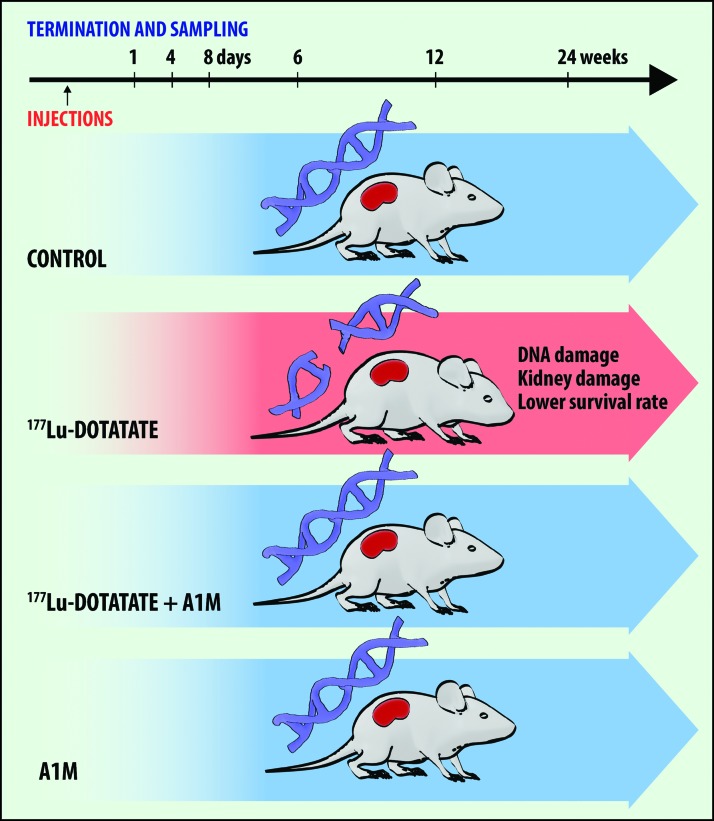
**Overview of design, groups, and results of the experiment presented in this article.** Four groups of nude female BALB/c mice received different injections: (i) control (PBS), (ii) ^177^Lu-DOTATATE, (iii) ^177^Lu-DOTATATE+A1M, and (iv) A1M. Animals from each group were terminated and sampled at six various time points after injection: 1 day, 4 days, 8 days, 6 weeks, 12 weeks, and 24 weeks. Injection of ^177^Lu-DOTATATE resulted in significant formation of DNA breaks, kidney damage (structural lesions and compromised renal function), and lower survival rate. Coadministration with A1M resulted in less DNA damage, renal function impairment, and fewer radiation-related deaths. A1M, α1-microglobulin; ^177^Lu, lutetium 177; PBS, phosphate-buffered saline. Color images are available online.

**FIG. 2.**
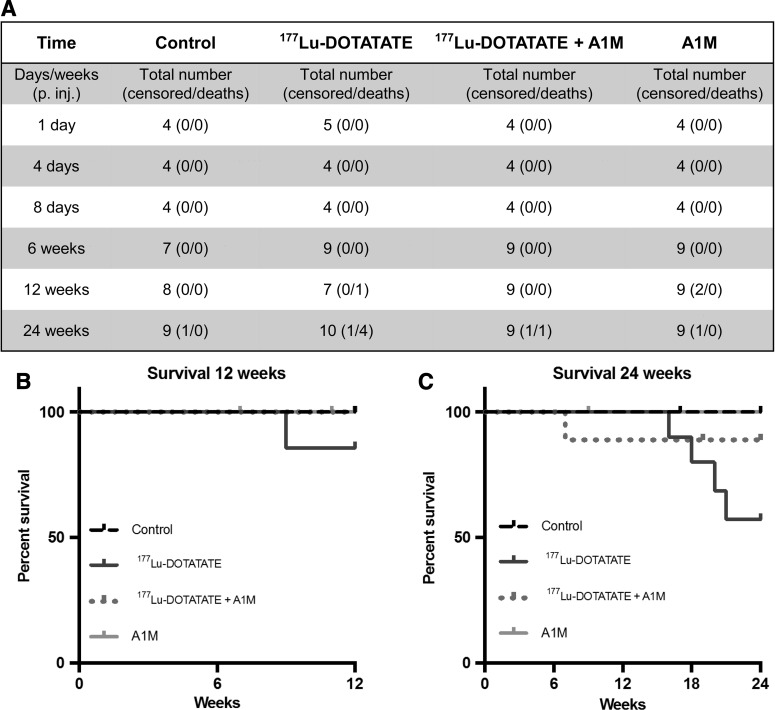
**Coadministration of A1M with ^177^Lu-DOTATATE increased survival.** Table presents total number of animals in all groups at all time points **(A)**. Number of animal deaths and animals removed from study (censored) are also presented. **(B, C)** Kaplan–Meier survival curve of control, ^177^Lu-DOTATATE, ^177^Lu-DOTATATE+A1M, and A1M groups. The experiment for the different groups ran 12 **(B)**, respectively, 24 **(C)** weeks postinjections. Censored animals are marked in the graph at the time point of the censoring. The causes of death of censored mice were weight loss, skin lesions, and swollen joints. Deaths were classified as radiation related when any of the following were found: swollen liver, swollen kidneys, swollen spleen, pathological spleen, and pathological liver. *p* = 0.0397; control mice compared with ^177^Lu-DOTATATE mice and *p* = 0.2731 (n.s.); ^177^Lu-DOTATATE mice compared with ^177^Lu-DOTATATE+A1M mice in the 24-week groups. (Mantel–Cox log-rank test).

Figure 1 shows the experimental design and groups. In the groups up to 6 weeks after ^177^Lu-DOTATATE injection, no radiation-related deaths occurred (Fig. 2A). After 12 weeks, one radiation-related death had occurred in the ^177^Lu-DOTATATE group and none in the other groups (Fig. 2B). After 24 weeks, the rate of survival in the ^177^Lu-DOTATATE injection group was 57%, significantly lower than in the control group (Fig. 2C). Coadministration of A1M increased the survival rate almost twofold, bringing the total overall survival rate up to 89%, which is similar to the control group and the group receiving A1M injections only (100%). The most common observations seen during postmortem autopsy in the ^177^Lu-DOTATATE therapy group were a swollen liver and/or spleen and atrophy of kidneys. In animals receiving coadministration of A1M, in addition to ^177^Lu-DOTATATE, only one pathological observation was recorded in one individual, a swollen spleen.

### Detection of γH2AX-labeled nuclear foci in kidneys

**FIG. 3.**
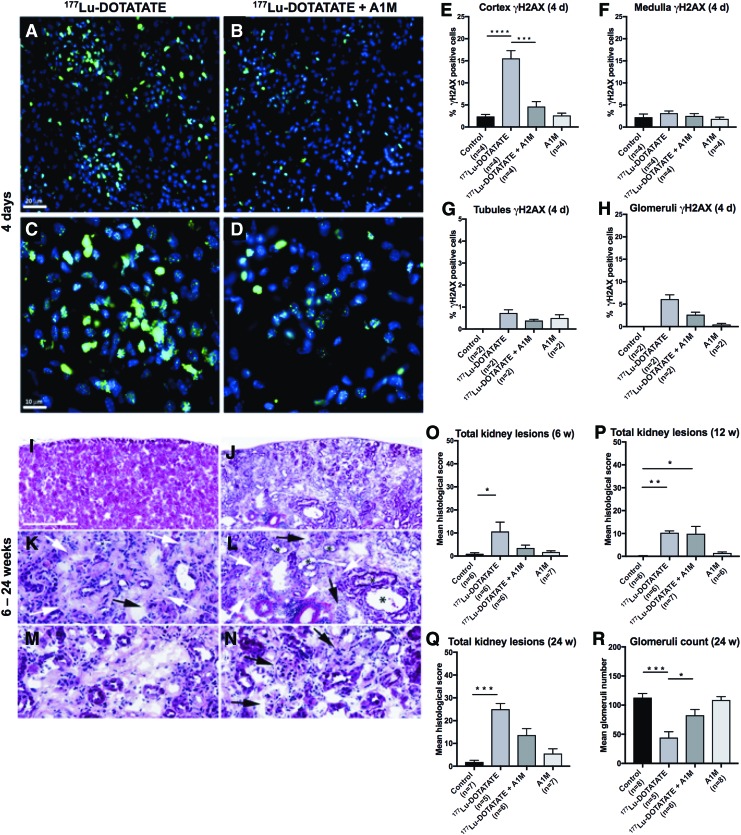
**A1M decreases double-strand DNA breaks in the kidneys and reduces kidney damage after injection with ^177^Lu-DOTATATE. (A–H)** Immunofluorescence labeling of the DNA damage marker γH2AX in kidney sections from the four groups. **(A–D)** Representative images of γH2AX immunofluorescence-labeled foci (*green*) and cell nuclei (*blue*) in kidney glomeruli and surrounding tubuli of kidney sections, 4 days after injections of ^177^Lu-DOTATATE **(A, C)** or ^177^Lu-DOTATATE+A1M **(B, D)**. Scale bar in **(A)** indicates 20 μm, applicable in **(A, B)**, and in–(**C)** indicates 10 μm, applicable in **(C, D)**. **(E–H)** Percentage of γH2AX-positive cells in the four groups in cortex **(E)**, medulla **(F)**, cortical tubules **(G)**, and glomeruli **(H)** 4 days postinjections. **(I–R)** Histopathological findings in the kidney from a ^177^Lu-DOTATATE-injected nude female BALB/c mouse (24 weeks) showing advanced stage of nephropathy **(J)** compared with the corresponding cortical region in a normal kidney from a control animal **(I)**. **(K–N)** depict higher magnification fields from **(J)** showing areas of fibrosis (*white arrows*), glomerular changes, including glomerular degeneration and atrophy, and glomerulosclerosis (*black arrows*). Inflammation (*white arrowhead*) and tubular dilation (*asterisk*) are also depicted. The *pale pink* areas depict tubular necrosis and degeneration found in ^177^Lu-DOTATATE-injected animals **(M)**. Scale bar in **(I)** represents 500 μm in **(I, J)**, 125 μm in **(K, M, N)**, and 250 μm in **(L)**. **(O–R)** Total scores of kidney lesions found in the four mouse groups. Eight different lesions were separately scored (0–4 severity; shown in Supplementary Fig. S1) and totaled for each animal. The mean of each group is presented at **(O)** 6 weeks, **(P)** 12 weeks, and **(Q)** 24 weeks postinjections. The glomerular count of morphologically defined normal glomerular units is shown as 24 weeks postinjections **(R)**. Assessments were performed in hematoxylin–eosin-stained kidney sections (10 μm). Statistical comparison was made between control and ^177^Lu-DOTATATE, control and ^177^Lu-DOTATATE+A1M, respectively, and ^177^Lu-DOTATATE and ^177^Lu-DOTATATE+A1M **(E, F**, **O–R)**. Only significant differences are presented in the figure. No statistical calculations are presented in **(G, H)** due to the low number of samples. Values are presented as mean ± SEM. Differences in groups were analyzed using one-way ANOVA with *post hoc* Tukey's test **(E, F**, **R)** and Kruskal–Wallis test with *post hoc* Dunn's test for histological scoring **(O–Q)**. *p < 0.05, **p < 0.01, ***p < 0.001, ****p < 0.0001. γH2AX, H2AX phosphorylated on serine 139; ANOVA, analysis of variance. Color images are available online.

The presence of cell nuclei expressing H2AX phosphorylated on serine 139 (γH2AX), a biomarker for repair of DNA double-strand break (DSB), was evaluated in sagittal sections of renal tissue 1, 4, and 8 days postinjection. Immunofluorescence (IF) detection of γH2AX revealed a distinct labeling of nuclear foci in the cortex of ^177^Lu-DOTATATE-injected animals and a higher number of γH2AX-labeled cell nuclei compared with that of control animals and animals coadministered with A1M (Fig. 3A–D).

γH2AX foci were detected on all days with maximal occurrence 4 days postinjection. The distribution of foci in the different groups and in renal compartments 4 days postinjection is presented in Figure 3E–H. The highest concentration of γH2AX-immunolabeled foci was seen in the renal cortex and there were fewer in the medulla. A majority of γH2AX-labeled foci were located in glomerular cells, while fewer were detected in tubules. In animals receiving coadministration of A1M, lower formation of γH2AX foci was observed compared with animals receiving only ^177^Lu-DOTATATE, and this difference was significant in the cortex with similar tendencies detected in the medulla, tubules, and glomeruli. These differences were detected at all the three time points evaluated (4 days postinjection, illustrated in Fig. 3).

### Kidney histopathology

Histological evaluation of kidney tissue 6, 12, and 24 weeks postinjection is summarized in Figure 3I–R and a detailed description of the scoring and results are presented as Supplementary Data. Pathological findings were separately scored (0–4 severity) for each lesion (8), totaled for each animal, and are presented as the mean of each group. In summary, the total number of pathological findings observed following ^177^Lu-DOTATATE exposure increased over time and was significantly lower following coadministration of A1M. Figure 3I–N exemplifies findings in one animal 24 weeks postinjection of ^177^Lu-DOTATATE.

Figure 3O–Q shows the accumulated kidney lesion scores in each of the four groups after 6, 12, and 24 weeks. Six weeks after ^177^Lu-DOTATATE injection, pathological findings were observed in several of the animals, including fibrosis in the kidney cortex and tubular and glomerular lesions (Fig. 3O). Severity grades for these findings ranged from 1 to 4. In contrast, findings in animals coadministered with A1M consisted of relatively mild tubular changes and fibrosis in the cortex and with lower severity grades (1 or 2).

At 12 weeks, the group receiving only ^177^Lu-DOTATATE displayed histopathological findings similar to those observed at 6 weeks. In all animals of this group (*n* = 6), atrophy was detected in the cortex and medulla (Fig. 3P). The most frequent findings with high severity were fibrosis in the cortex together with glomerular and tubular findings, mainly with severity grades ranging from 2 to 3. Animals coadministered with A1M displayed a high variation between individuals. Two animals showed severe findings (grades 3 to 4), one animal showed no lesions at all, and in the remaining four animals, only low-grade (1–2) findings of fibrosis in the cortex and medulla, glomerular and tubular findings, and inflammatory changes were observed.

At 24 weeks postinjection of ^177^Lu-DOTATATE (*n* = 5), animals displayed more frequent and pronounced lesions compared with other groups (Fig. 3Q). Coadministration with A1M resulted in less frequently observed damage compared with animals receiving only ^177^Lu-DOTATATE in all (*n* = 8) the examined lesions (Supplementary Fig. S1A–H). In animals injected with only A1M, the findings were few, mainly low grade (1–2), and most frequently consisting of inflammatory and tubular findings. At 24 weeks, glomerulus counting showed a significant decrease in the ^177^Lu-DOTATATE group compared with animals coadministered with A1M (Fig. 3R).

### Effects on gene expression in kidneys and liver

**FIG. 4.**
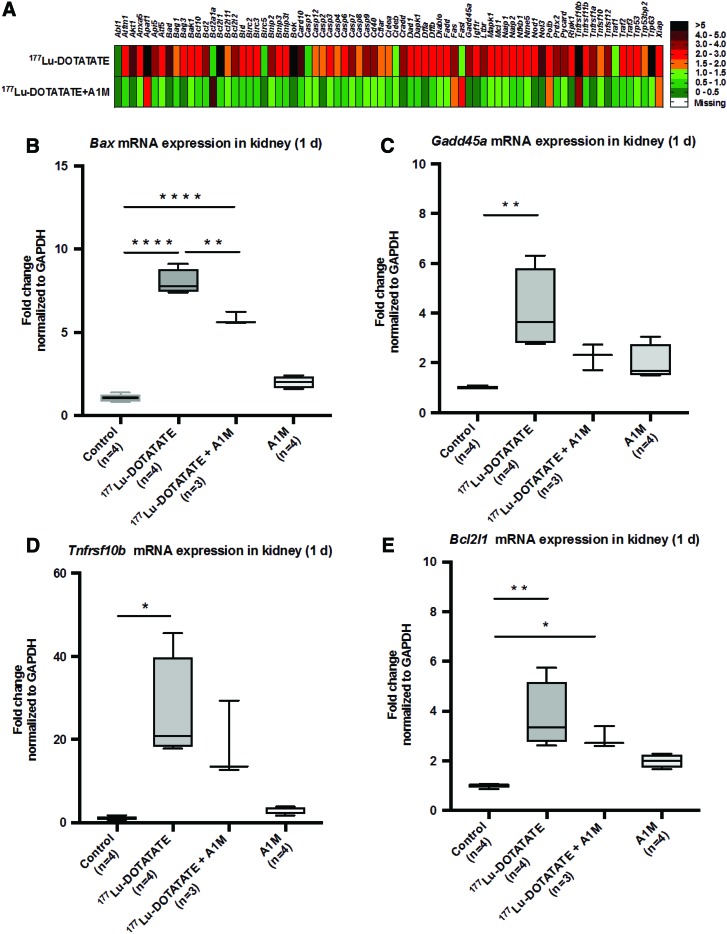
**^177^Lu-DOTATATE injections in nude BALB/c mice change gene expression of genes associated with apoptosis. (A)** Messenger RNA expression of an apoptosis-related gene array in pooled renal tissue from all individuals (*n* = 4 control and ^177^Lu-DOTATATE+A1M and *n* = 5 ^177^Lu-DOTATATE) terminated 1 day postinjection, normalized to expression of the *β*2*-microglobulin* gene, and expressed as fold change relative to the control group, where fold change 1 equals the control group. *Green* indicates a fold change of 0–1.5, *orange* 1.5–2.0, *red* 2.0–5.0, and *black* above 5. **(B–E)** Analysis of mRNA expression of *Bax* **(B)**, *Gadd45a* **(C)**, *Tnfrsf10b* **(D)**, and *Bcl2l1* **(E)** at the individual level, 1 day postinjections, normalized to expression of *GAPDH*. Statistical comparison was made between control and ^177^Lu-DOTATATE, control and ^177^Lu-DOTATATE+A1M, respectively, and ^177^Lu-DOTATATE and ^177^Lu-DOTATATE+A1M. Only significant differences are presented in the figure. Values, normalized against the control group, are presented as mean fold change with *whiskers* indicating 5 to 95 percentiles. Outliers were removed using the ROUT method (*Q* = 1%) **(B–E)**. Differences in groups were analyzed using one-way ANOVA with *post hoc* Tukey's test **(B–E)**. *p < 0.05, **p < 0.01, ****p < 0.0001. *Bax*, bcl-2-like protein 4; *Bcl211*, Bcl-2-like 1; *Gadd45a*, growth arrest and DNA damage-inducible protein alpha; *GAPDH*, glyceraldehyde 3-phosphate dehydrogenase; mRNA, messenger RNA; *Tnfrsf10b*, tumor necrosis factor receptor superfamily member 10B. Color images are available online.

The expression of genes involved in apoptosis induction and regulation was evaluated in short-term (day 1–8) renal tissues using a profiler array on pooled samples from all individuals in each group (*n* = 4 in control and ^177^Lu-DOTATATE+A1M groups and *n* = 5 in the ^177^Lu-DOTATATE group). An increased expression of almost all investigated genes was seen in animals receiving ^177^Lu-DOTATATE 1 day after the injection (Fig. 4A) compared with control animals. In contrast, almost all of these genes remained unchanged, or only slightly increased, in animals receiving coadministration of A1M compared with control animals. Evaluation at 8 days after injection resulted in less pronounced differences between all groups (not shown). To further evaluate the observed changes at day 1, expression levels of four of the most affected genes, bcl-2-like protein 4 (*Bax*), tumor necrosis factor receptor superfamily member 10B (*TnfRsf10b*), Bcl-2-like 1 (*Bcl211*), and growth arrest and DNA damage-inducible protein alpha (*Gadd45a*), were analyzed at an individual level with specific real-time quantitative PCR (qPCR) analysis.

An increased expression, compared with control animals, of all the evaluated genes was seen following exposure to ^177^Lu-DOTATATE (Fig. 4B–E). Coadministration of A1M displayed significantly reduced messenger RNA (mRNA) expression, compared with the ^177^Lu-DOTATATE animals, of *Bax* and a clear trend toward reduced expression of all other genes evaluated.

**FIG. 5.**
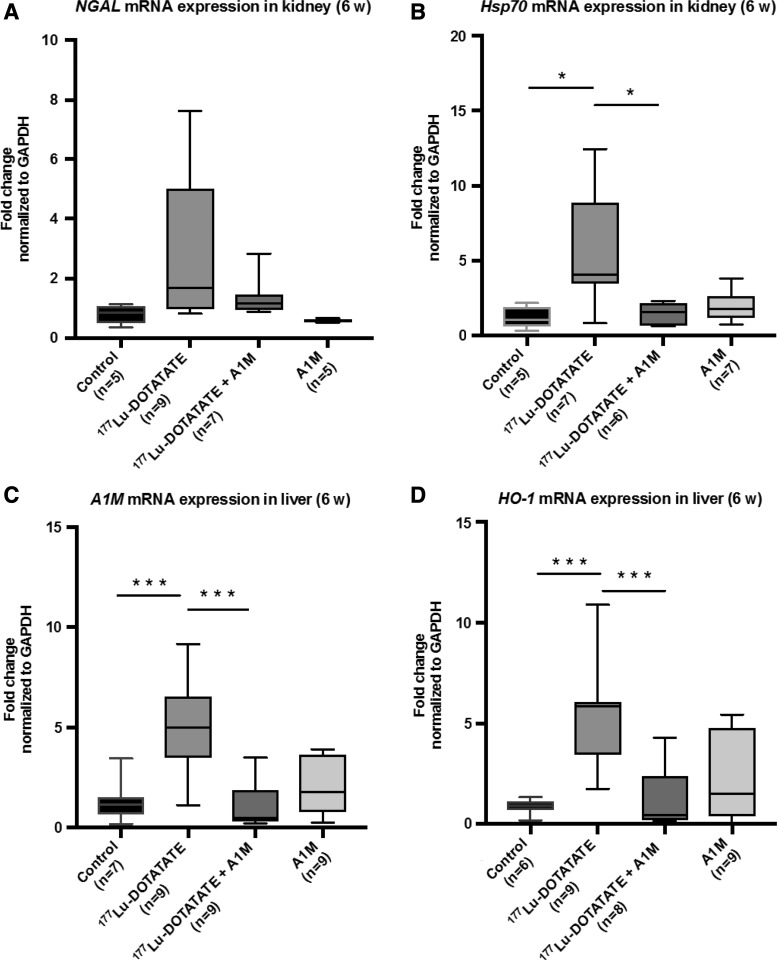
**^177^Lu-DOTATATE injections in nude BALB/c mice change gene expression of genes associated with inflammation and oxidative stress. (A–D)** Analysis of mRNA expression of *NGAL* **(A)**, *Hsp70* **(B)** in kidney tissue, and *A1M* **(C)** and *HO-1* **(D)** in the liver at the individual level, 6 weeks postinjection. Statistical comparison was made between control and ^177^Lu-DOTATATE, control and ^177^Lu-DOTATATE+A1M, respectively, and ^177^Lu-DOTATATE and ^177^Lu-DOTATATE+A1M. Only significant differences are presented in the figure. Values, normalized against the control group, are presented as mean fold change normalized to *GAPDH* expression values, with *whiskers* indicating 5 to 95 percentiles. Outliers were removed using the ROUT method (*Q* = 1%). Differences in groups were analyzed using one-way ANOVA with *post hoc* Tukey's test. *p < 0.05, ***p < 0.001. *HO-1*, heme oxygenase 1; *Hsp70*, heat shock protein 70; *NGAL*, neutrophil gelatinase-associated lipocalin.

In addition to short-term evaluation, the mRNA expression levels of neutrophil gelatinase-associated lipocalin (*NGAL*) and heat shock protein 70 (*Hsp70*) were evaluated in renal tissue at 6 weeks following ^177^Lu-DOTATATE injection (Fig. 5A, B). Animals receiving ^177^Lu-DOTATATE displayed a significant upregulation of *Hsp70* and a trend toward upregulation of *NGAL*, although not significant, compared with the control group. Coadministration of A1M resulted in significantly reduced mRNA expression of *Hsp70*, compared with the ^177^Lu-DOTATATE group, and a clear trend toward normalizing the expression of *NGAL*.

Furthermore, mRNA expression levels of heme oxygenase 1 (*HO-1*) and *A1M* were evaluated in liver tissue at 6 weeks following ^177^Lu-DOTATATE injections (Fig. 5C, D). There was a significant upregulation of both genes in animals receiving ^177^Lu-DOTATATE compared with the control group. In animals coadministered with A1M, a significantly decreased response could be observed for both *HO-1* and *A1M* compared with the ^177^Lu-DOTATATE group.

### Biological markers associated with renal function

**FIG. 6.**
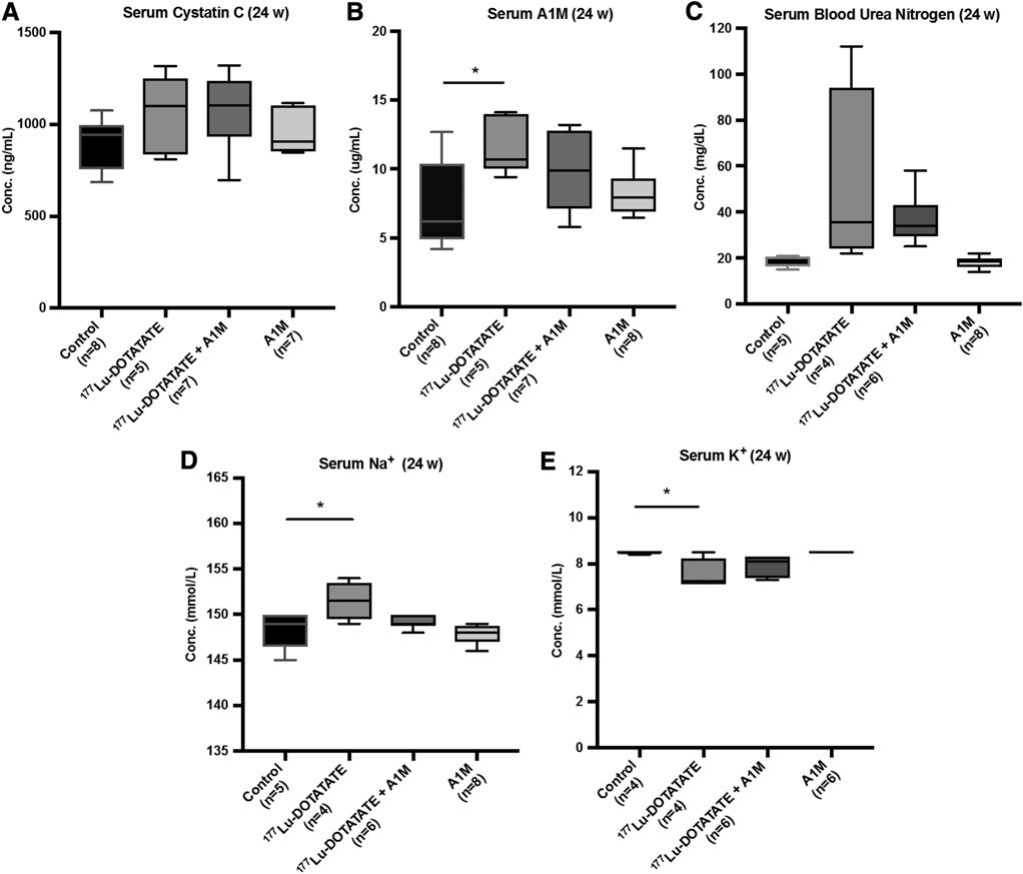
**^177^Lu-DOTATATE injections in nude BALB/c mice affect serum levels of markers associated with kidney function.** Scatter plots depict **(A)** serum cystatin C, **(B)** A1M, **(C)** BUN, **(D)** Na^+^, and **(E)** K^+^ levels 24 weeks postinjections measured by **(A)** ELISA, **(B)** radioimmunoassay, or **(C–E)** the VetScan VS2 chemistry analyzer in the four mouse groups. Statistical comparison was made between control and ^177^Lu-DOTATATE, control and ^177^Lu-DOTATATE+A1M, respectively, and ^177^Lu-DOTATATE and ^177^Lu-DOTATATE+A1M. Only significant differences are presented in the figure. Values are presented as mean with *whiskers* indicating 5 to 95 percentiles. Outliers were removed using the ROUT method (*Q* = 1%). Differences in groups were analyzed using one-way ANOVA with *post hoc* Tukey's test. *p < 0.05. K^+^, potassium ion; Na^+^, sodium ion.

Urinary albumin and serum levels of cystatin C, A1M, blood urea nitrogen (BUN), sodium ion (Na^+^), and potassium ion (K^+^) were analyzed as markers of renal function and damage. Serum cystatin C levels increased with ^177^Lu-DOTATATE injections, which were also seen in the animals receiving coadministration of A1M; however, these changes were not significantly different from control animals (Fig. 6A).

A significant increase in serum A1M levels was observed 24 weeks (Fig. 6B), but not 6 and 12 weeks, following ^177^Lu-DOTATATE injections compared with the control group. In the group receiving coadministration of A1M, this increase was mitigated. BUN levels were not significantly different between groups, although there is a tendency to an increase following ^177^Lu-DOTATATE injections, less so in animals receiving coadministration of A1M (Fig. 6C). Serum Na^+^ and K^+^ concentrations are significantly increased and decreased, respectively, following the ^177^Lu-DOTATATE injection; however, these changes were not seen in animals receiving coadministration of A1M (Fig. 6D, E).

**FIG. 7.**
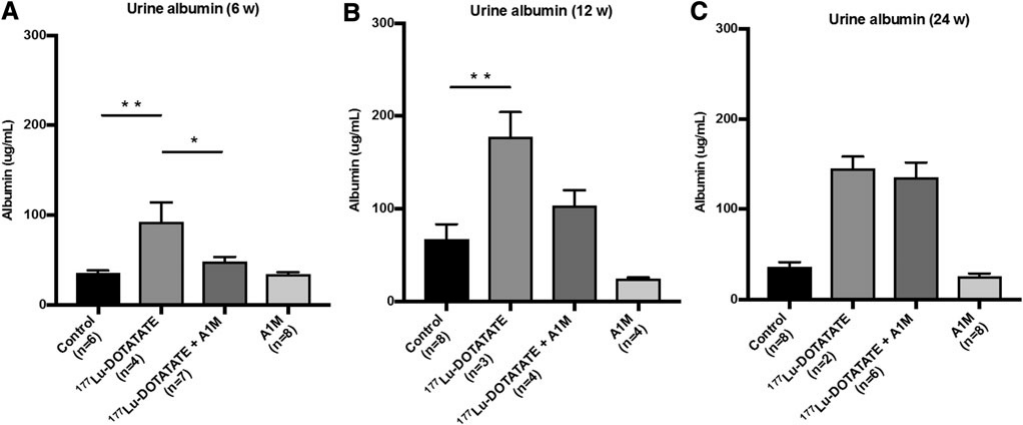
**^177^Lu-DOTATATE injections in nude BALB/c mice affect urine albumin levels, a marker of proteinuria.** Urine level of the functional marker albumin in BALB/c nude mice **(A)** 6 weeks, **(B)** 12 weeks, and **(C)** 24 weeks postinjections, measured by ELISA. Statistical comparison was made between control and ^177^Lu-DOTATATE, control and ^177^Lu-DOTATATE+A1M, respectively, and ^177^Lu-DOTATATE and ^177^Lu-DOTATATE+A1M. Only significant differences are presented in the figure. No statistical comparison is presented in the 24-week animals due to low number of samples from the ^177^Lu-DOTATATE animals (*n* = 2). Values are presented as mean ± SEM. Outliers were removed using the ROUT method (*Q* = 1%). Differences in groups were analyzed using one-way ANOVA with *post hoc* Tukey's test. *p < 0.05, **p < 0.01.

In all long-term animals, urine albumin concentrations were significantly increased following ^177^Lu-DOTATATE exposure, peaking at 12 weeks (Fig. 7A–C). At all time points, except at 24 weeks, coadministration of A1M consistently mitigated the increase, most notably at 6 weeks. However, it should be noted that five of the animals in the ^177^Lu-DOTATATE group were terminated before reaching 24 weeks due to bad health. Thus, only the five most healthy animals were included in the analysis. Of the remaining animals, only two urine samples were collected, therefore no statistical comparison is made in the groups 24 weeks postinjections.

Due to low amount of urine, creatinine measurements could not be performed to establish an albumin-to-creatinine ratio. However, in a new experiment, urine albumin was corrected for with creatinine levels and had a decreasing trend with coadministration of A1M compared with animals receiving only ^177^Lu-DOTATATE (Supplementary Fig. S2).

## Discussion

In this work, we have shown that human antioxidation and radical scavenger protein A1M protects kidneys from ^177^Lu-DOTATATE irradiation damage. Thus, coadministration of A1M with the radiopharmaceutical resulted in a clear reduction of short-time nephrotoxic effects on DNA and gene expression levels. The kidneys are late-responding organs, and kidney impairment as a result of PRRT can take years to develop in humans and mice (4, 28). Therefore, we also investigated long-term effects and could show significant protection of kidney morphology and function by A1M coadministration.

Human A1M has been shown to have physiological, antioxidant protective functions by using a combination of reduction, radical-binding, and heme-binding molecular mechanisms (1a). It has previously been shown that A1M inhibits the so-called bystander effect of ionizing particle irradiation *in vitro*, that is, spreading of cell death/damage from targeted cells to surrounding nonirradiated cells, mainly by inhibition of oxidative stress in bystander cells (21, 25). This is consistent with a protection model where A1M primarily targets indirect oxidative irradiation effects rather than direct effects of the radionuclide. The most likely molecular mechanisms of A1M for protection of kidney tissue are therefore (i) scavenging of reactive oxygen species (ROS) and radicals generated by interactions between the emitted particles from ^177^Lu and cell and tissue constituents and (ii) reduction of downstream oxidation lesions on cell and matrix components.

When DSBs in DNA occur, the H2AX histone is phosphorylated into γH2AX (14), forming quantifiable γH2AX foci that are frequently used to evaluate DNA damage. In this study, we observed that ^177^Lu-DOTATATE injections induced a significant increase in DSBs, which was reversed by A1M coadministration (Fig. 3). A higher number of DSB foci were seen in the cortex compared with the medulla. This should be expected since a much higher dose of injected ^177^Lu-DOTATATE was seen in the cortex compared with the medulla (1). Furthermore, more DSB foci were seen in glomeruli compared with tubuli. In a study parallel to this, the absorbed dose rate was shown to be higher in tubules (Ahlstedt *et al.*, manuscript in preparation), suggesting that glomerular tissue is more radiosensitive than the tubular epithelium.

Our previous pharmacokinetic analysis of A1M in kidneys showed that maximal concentration of the injected protein occurred 10 min after injection and that more than 99% had been cleared after 24 h (1). This strongly suggests that most of the irradiation damage as well as protection by A1M occurs during the first hours after injection. Yet, the induction of γH2AX foci was at its maximum at 4 days after the radioactivity injection, suggesting that most of this DNA damage is a result of downstream events rather than the irradiation itself. Likewise, more than 80% of the γH2AX induction after 4 days was inhibited by A1M, lending further support to the view that a majority of these DSBs are secondary effects of free radical-induced oxidative stress caused by high absorbed doses of ^177^Lu-DOTATATE during the first hours.

Histopathological scoring of kidney damage revealed distinct differences between the groups and relatively low individual variations between animals within the groups (Fig. 3). The histological damage was most pronounced in ^177^Lu-DOTATATE-injected animals after 24 weeks. Although not significant, there was a clear trend in all eight lesions examined that showed ameliorated damage after coadministration with A1M (Supplementary Fig. S1A–H).

Furthermore, the glomerulus count was reduced to half after 24 weeks following ^177^Lu-DOTATATE injections and was significantly reversed in the A1M-coadministered group. Together, this implies that there is continuous deterioration of kidneys after ^177^Lu-DOTATATE exposure, which is ameliorated with coadministration of A1M. The few low-grade findings in control groups, most notably in the 24-week group, could be explained by inflammatory responses, which might influence kidney pathology.

Of note, the histopathological evaluation in this study was performed according to standard criteria used for toxicological pathology (7, 18), where atrophy, fibrosis, deformed glomerular, tubular and/or collecting duct morphology, necrosis, and inflammation were evaluated (see Supplementary Data for more detailed information). Additional studies on more specific processes, such as apoptosis (using TUNEL staining), were not performed since a detailed evaluation of pathological mechanisms in kidneys after ^177^Lu-DOTATATE exposure was not within the scope of this investigation.

To investigate kidney function, several biomarkers were evaluated. Plasma cystatin C and BUN levels, both associated with a reduced glomerular filtration rate, did not show statistically significant differences between the groups (Fig. 6A, C). An indication of impaired renal function, however, was the significant increase in serum A1M levels in animals injected with ^177^Lu-DOTATATE (Fig. 6B) (11). The mean value was repressed in the group coinfused with A1M, but the difference compared with the group injected with ^177^Lu-DOTATATE was not statistically significant. Sodium and potassium levels were significantly shifted in the ^177^Lu-DOTATATE group compared with the control group (Fig. 6D, E); this electrolyte imbalance implies disturbed tubular function. However, the intake of water and food was not monitored, so these changes could be attributed to differences in intake.

Significantly increased albumin in the urine, a marker of renal pathology, was observed in the ^177^Lu-DOTATATE group at 6, 12, and 24 weeks and was reduced with coadministration of A1M (Fig. 7A–C). Proteinuria may result from glomerular damage and/or low reabsorption in proximal tubules (12). These results suggest that ^177^Lu-DOTATATE injections result in development of kidney dysfunction, which is slowed by coadministration of A1M.

The mRNA expression in renal tissue was investigated by screening of genes related to apoptosis and cell stress response using an apoptosis-related gene array, followed by a more detailed evaluation of selected genes. The selected genes, *Bax* (8), *TnfRsf10b* (17), *Bcl2l1* (6), and *Gadd45a* (31), all involved in apoptotic pathways, were upregulated, suggesting increased cell death 1 day after injection of ^177^Lu-DOTATATE (Fig. 4B–E). In the A1M-coadministered group, *Bax* was significantly reduced and the other genes had tendencies to decreased expression. At 6 weeks postinjection, the trend toward increase in *NGAL* mRNA expression, in the ^177^Lu-DOTATATE animals, supports histopathological findings in terms of renal tissue scarring and specific tubular findings—a sign of acute kidney damage (30) (Fig. 5A). Hsp-70, a protein related to protein folding homeostasis (24), showed a significant increase of mRNA levels in animals injected with ^177^Lu-DOTATATE. This response was almost completely inhibited by A1M coadministration (Fig. 5B).

The mRNA expression levels of *HO-1* and *A1M* in the liver were both significantly upregulated in the ^177^Lu-DOTATATE group compared with all other groups (Fig. 5C, D) after 6 weeks. HO-1 is associated with cytoprotective functions and inflammatory modulation and is upregulated not only in the presence of its substrate heme (23) but also in response to other oxidative stress-inducing factors, for example, ROS (13). A1M is mainly produced in the liver and is upregulated in the presence of hemoglobin and ROS (20).

Upregulation of these genes in the liver suggests a global physical stress response after ^177^Lu-DOTATATE injections, which is reduced to control levels with A1M coadministration. Although a change in mRNA expression does not always lead to a changed protein level, upregulation of *HO-1* and *A1M* genes in the liver may contribute to protection of kidneys *via* increased secretion to the bloodstream.

Side effects of ^177^Lu-DOTATATE therapy, besides kidney injury and the liver response shown here, include adverse effects on spleen, bone marrow, and blood cells (4). We have not studied those effects in this work, but further studies of protective effects of A1M on blood cells may be motivated considering that binding of A1M to red and white blood cells has been reported (22, 33).

To be able to use A1M as a radiation protection agent in clinical PRRT, it is essential that infusion of A1M does not protect the tumor tissue itself, that is, A1M allows full therapeutic effect of ^177^Lu-DOTATATE on tumors. Protection of kidneys by A1M, as shown here, is probably due to specific localization of the protein to this organ (1). It is not expected that A1M would be localized to neuroendocrine tumor tissue after infusion since there are no reports of specific receptors or other uptake mechanisms in such tumor cells. Therefore, we do not expect that A1M would affect tumor therapy negatively in PRRT. However, this must be shown experimentally, and the uptake of A1M in tumor tissue and the influence of A1M on tumor therapy, in a mouse model, are being investigated in a separate project running parallel to this study.

In summary, the results of this study have demonstrated that coadministration of A1M inhibits ^177^Lu-DOTATATE-induced DNA damage, kidney structure damage, stress response, reduced glomerular filtration rate, and proteinuria up to 6 months after injections. This suggests that A1M can be used during PRRT treatment to improve the clinical outcome by increasing tumor control and increasing the number of patients who can receive PRRT treatment.

The standard protocol today is aimed to minimize kidney damage by (i) limiting the therapeutically administered activity and thus the absorbed dose in kidneys and (ii) infusion of amino acids to block tubular uptake of radionuclide peptides. The first protective measure limits treatment effects and the latter procedure—infusion of amino acids—has strong negative effects on patient well-being (nausea). Moreover, the treatment is performed in fractions until the maximum tolerable absorbed dose in kidneys is reached. Coadministration of A1M could therefore potentially improve PRRT by allowing higher treatment activities, increased number of fractions, and, finally, exclusion of amino acid infusions.

## Methods

### Recombinant human A1M

Recombinant human A1M (A1M), with an N-terminal His-tag, was expressed, purified, and refolded from *Escherichia coli* cultures as described by Kwasek *et al.* (15), with modifications as outlined (1).

### Radiopharmaceutical analysis

Radiolabeling of the DOTATATE peptide with lutetium (^177^Lu, *E*_max_ = 0.5 MeV, half-life = 6.73 days) chloride (LuMark, IDB, Holland) was performed at Lund University Hospital (Lund, Sweden). Quality control of the resulting ^177^Lu-DOTATATE conjugate was performed at the Lund University Radionuclide Centre (Lund, Sweden).

### Animal studies

For the short-term study groups, a total of 48 female nude BALB/c mice (Charles River, Germany) at the age of 8 weeks were used. To ensure a measurable effect on kidneys as well as to minimize any discrepancies that might occur due to some difference in age and weight, animals were injected with 150 MBq of ^177^Lu-DOTATATE (29). Two groups (*n* = 4, except ^177^Lu-DOTATATE 1 day *n* = 5) received ^177^Lu-DOTATATE (150 MBq, 75 μL, 42 ± 2.3 Gy to the kidneys) by i.v. injection in the tail vein, immediately followed by either A1M (7 mg/kg, 75 μL, 10 m*M* Tris-HCl) or phosphate-buffered saline (PBS). A third group (*n* = 4) was injected with A1M (7 mg/kg, 75 μL, 10 m*M* Tris-HCl), followed by PBS, and last, a control group (*n* = 4) received dual injections of PBS. Animals were sacrificed 1, 4, and 8 days postinjections.

For the long-term groups, a total of 104 female nude BALB/c mice (Charles River) at the age of 13–16 weeks were used. As above, two groups (*n* = 9, except ^177^Lu-DOTATATE 12 weeks *n* = 7 and 24 weeks *n* = 10) received ^177^Lu-DOTATATE (150 MBq, 75 μL, 42 ± 2.3 Gy to the kidneys), followed by either A1M (7 mg/kg, 75 μL, 10 m*M* Tris-HCl) or PBS. The third group (*n* = 9) was injected with A1M (7 mg/kg, 75 μL, 10 m*M* Tris-HCl), followed by PBS, and the control group (6 weeks *n* = 7, 12 weeks *n* = 8, and 24 weeks *n* = 9) with two PBS injections. Animals were sacrificed 6, 12, and 24 weeks postinjections.

Before euthanasia, urine was collected from each animal and put on dry ice. Following termination, blood was collected *via* heart puncture. One kidney and the liver were put on dry ice for mRNA and protein extraction. The second kidney was harvested for immunohistochemistry (IHC) and IF labeling and immediately frozen in Tissue-Tek embedding medium (Sakura Finetek).

All animal experiments were conducted in compliance with the national legislation on laboratory animals' protection and with the approval of the Ethics Committee for Animal Research (Lund, Sweden, project no. M50-16).

### Survival analysis

All the mice in the long-term groups were included in the survival analysis. Animals were censored at the end point of the experiment (6, 12, and 24 weeks) when they were sacrificed for further analysis. Survival analysis was performed using the Kaplan–Meier estimator for all groups. Comparison among control, ^177^Lu-DOTATATE, and ^177^Lu-DOTATATE+A1M groups was analyzed using the Mantel–Cox log-rank test. Humane end points of the survival study were weight loss, skin lesions, swollen joints, and/or general physical decline (signs of distress or pain). If findings during examination postmortem included radiation-related damage (swollen liver, swollen kidneys, swollen spleen, pathological spleen, and pathological liver), these animals were included in the survival analysis, otherwise they were censored.

### IF labeling of γH2AX and histological scoring

Serial sectioning of frozen kidney samples was performed in a cryostat (Leica CM1950; Leica Microsystems AB, Sweden). Adjacent sections (10-μm thick) were collected on microscope slides (SuperFrost Plus slides; Merck, Darmstadt, Germany), from the middle portion of the kidney, yielding three to four sections per slide. For immunolabeling, sections were fixed in 100% acetone (Histolab, Gothenburg, Sweden) for 5 min and rinsed in PBS twice for 3 min.

For IF labeling, sections were first blocked with normal donkey serum, followed by incubation in rabbit anti-γH2AX (Thermo Fisher) diluted 1:800 for 16 h at 4°C. After rinsing in PBS, sections were incubated with secondary antibodies, donkey anti-rabbit IgG conjugated with AlexaFlour488 (Jackson Immunoresearch, West Grove, PA) in a dilution of 1:400 (in PBS), for 30 min at room temperature. Following rinses in PBS, cell nuclear counterstaining was performed with 4′,6-diamidino-2-phenylindole (DAPI; Invitrogen, Thermo Fischer, Sci, Inc.). After PBS rinsing, sections were mounted in Fluoroshield mounting media (Abcam, Cambridge, United Kingdom).

Control IF experiments with only secondary antibodies were performed, showing virtually no labeling, except weak background autofluorescence, supporting a specific labeling of the used primary and secondary antibodies. In addition, antibody control sections were used to set the detector threshold used for visual analyses and for documented images used in digital image analysis.

Sections for IF were examined using microscopes equipped for epifluorescence analysis (Olympus IX73; Olympus, Hamburg, Germany). Images were digitally documented with b/w using the Olympus DP80 camera. Images were used for illustrations and digital image analysis.

For histological scoring, sectioning of frozen kidney samples was performed in a cryostat (10-μm thick). Adjacent sections were collected on microscope slides (SuperFrost plus slides; Merck) from the middle portion of the kidney, yielding three to four sections per slide. Sections were fixed with 4% formaldehyde and stained with hematoxylin (Mayers HTX Plus; Histolab), followed by dehydration in EtOH (70%). Sections were counterstained with eosin solution (Eosin B; Applichem, GmbH, Darmstadt, Germany) and dehydrated in EtOH (96% and 100%), followed by xylene (Histolab). Sections were mounted in mounting medium (Pertex; Histolab).

As a minimum, two hematoxylin–eosin-stained cryosections (sagittal view) of the kidney per animal were analyzed for histopathological assessments of lesions/damage in the medulla (*n* = 3 lesions) and cortex (*n* = 5 lesions) as well as the glomerulus count (Fig. 3I–N). All remaining animals in the 24-week ^177^Lu-DOTATATE group were analyzed (*n* = 5). In the other groups, depending on the available number of animals and tissue quality (*i.e.*, acceptable for analyses), six to seven animals were analyzed (*n* = 6 and *n* = 7). The individuals in groups with more than seven animals were randomly selected.

Evaluation and grading were performed according to the standard reference for nomenclature and diagnostic criteria in toxicological pathology (18) for the following eight lesions: (i) Atrophy of the cortex was recognized by decreased size or compression of cortex layers. (ii) Atrophy of the medulla was recognized by decreased size or compression of medulla layers. (iii) Interstitial fibrosis in the cortex was recognized by the presence of interstitial fibrotic structures and scarring. (iv) Interstitial fibrosis in the medulla was recognized by the presence of interstitial fibrotic structures and scarring. (v) Glomerular findings (cortex), which included atrophy and degeneration, were recognized by loss of glomeruli and deformed glomerular morphology.

(vi) Tubular findings in the cortex included degeneration, necrosis, dilation, cysts, and hyperplasia. Degenerative and/or necrotic tubuli were recognized by deformed tubular epithelial cells, that is, cells encompassing morphologic changes such as tinctorial change, vacuolation, blebbing, and loss of or defragmented nuclei. Tubular dilation was recognized by mild to moderate expansion of lumina lined by relatively normal epithelium—tubular cysts are a more severe manifestation of tubular dilation. Tubular hyperplasia was recognized by enlarged tubuli with proliferating epithelial cells, that is, the presence of more than a single cell layer. (vii) Inflammatory findings in the cortex were recognized by the presence of focal or multifocal accumulations of tissue-infiltrated (extravasated) inflammatory cells identified from cell morphology.

(viii) Collecting duct findings (medulla), that is, degeneration of collecting duct structures, were recognized by degrading collecting ducts with atypical epithelial cells. Morphological findings were graded on a 0–4 severity scale as follows: 0 = normal, 1 = minimal, 2 = mild, 3 = moderate, and 4 = marked, totaled for each animal, and presented as the group mean for each lesion. Total kidney lesions are all lesions (*n* = 8) totaled for each animal and presented as the group mean.

For the glomerulus count, only clearly visualized and viable glomerular formations were counted, as opposed to the damaged that were recorded above. Assessments were performed in one sagittal section at the center position of the kidney for each animal.

### Image analysis and development of counting algorithms

To accurately quantify γH2AX foci labeled by IHC and IF in tissue sections, an in-house algorithm was developed using ImageJ Macro language (IJM). The software was used to identify cell nuclei (hematoxylin or DAPI stained) and, within each detected nucleus, measure the area (in pixels) of individual and clustered γH2AX foci. To accurately separate areas of interest from background noise, threshold pixel values were iteratively derived by manually counting cell nuclei and foci in randomly chosen areas and comparing the outcome with the findings of the software. Reliability of the resulting threshold values was validated by further random testing and used for all subsequent quantifications of cell nuclei and γH2AX foci.

Two sections of renal tissue (per animal) were divided into subsections of the cortex (*n* = 6) and medulla (*n* = 3) and analyzed using the ImageJ software platform to apply the algorithm.^*^ Each subsection contained a minimum of 103 hematoxylin- or DAPI-labeled cell nuclei. In each subsection, the number of cell nuclei exhibiting γH2AX staining within the defined threshold values was determined, and results were expressed as the percentage cell nuclei exhibiting such γH2AX staining. The total area of γH2AX staining in each subsection was also determined and yielded closely correlated values.

### PCR profiler array and mRNA analysis of specific genes

The expression of 84 genes associated with regulation of apoptosis was investigated using pooled renal tissue samples from all animals in the 1- and 8-day groups. Total RNA was isolated from frozen renal tissue using the RNeasy Mini Kit (QIAGEN, Valencia, CA). Whole kidney samples were used for extraction. Extracted RNA was reverse-transcribed into cDNA using the RT2 First Strand Kit supplied by QIAGEN. The presence of mRNA was quantified and analyzed with the RT2 PCR profiler array specified for mouse apoptosis (cat. no. PAMM-012Z) using RT2 SYBR Green Fluor qPCR Mastermix (QIAGEN) and visualized in a heat map as fold change values relative to the control group after normalization against a control gene, β_2_*-microglobulin*, supplied in the kit.

Selected from the profiling array of apoptosis-related genes with particularly pronounced differences between control and ^177^Lu-DOTATATE groups, mRNA expression levels of *Bax*, *TnfRsf10b*, *Bcl2l1*, and *Gadd45a* were analyzed in all individuals at days 1, 4, and 8 using the PrimePCR SYBR Green Assay (Bio-Rad, Hercules, CA). All data were normalized against the control gene glyceraldehyde 3-phosphate dehydrogenase (*GAPDH*). RNA amplification was performed using thermocycling conditions as recommended by the manufacturer, starting with an initial polymerase inactivation step at 95°C for 2 min, followed by 40 cycles at 95°C for 30 s, 60°C for 1 min, and 72°C for 1 min (iCycler Thermal Cycler; Bio-Rad).

In addition, total RNA was isolated from frozen renal tissue and liver tissue from all individual animals in the 6-week group using the Nucleospin RNA/prot kit (Macherey-Nagel, Duren, Germany), followed by the RNeasy Mini Kit. Extracted RNA was reverse-transcribed into cDNA using the iScript cDNA synthesis kit (Bio-Rad). mRNA expression levels of *NGAL, HO-1*, and *Hsp70* were analyzed in renal tissue and *A1M* and *HO-1* in liver tissue and normalized against the control gene *GAPDH* using the iTaq Universal SYBR Green Supermix (Bio-Rad). RNA amplification was performed using thermocycling conditions as recommended by the manufacturer, starting with an initial polymerase inactivation step at 95°C for 3 min, followed by 40 cycles at 95°C for 5 s and 60°C for 30 s (CFX Connect Real-Time PCR Detection System; Bio-Rad).

### Functional markers

Albumin concentrations in urine were analyzed using a mouse albumin ELISA kit (Abcam) according to instructions provided by the manufacturer. Urine creatinine concentrations were analyzed using a colorimetric assay according to instructions from the manufacturer (Biovision, Milpitas, CA). Serum concentrations of cystatin C were analyzed using an ELISA kit (Mouse/Rat Cystatin C Quantikine ELISA Kit; R&D Systems, Minneapolis, MN) according to instructions provided by the manufacturer. Serum concentrations of BUN, K^+^, and Na^+^ were determined using a clinical chemistry analyzer, VetScan VS2 (Abaxis, Union City, CA), and the VetScan Comprehensive Diagnostic Profile reagent rotor according to instructions from the manufacturer.

A1M concentrations in serum were measured with radioimmunoassay, as previously described (9), using recombinant mouse A1M and rabbit antirecombinant mouse A1M prepared in our laboratory as described (26). Radiolabeling of A1M with iodine 125 was done using the chloramine-T method (10).

### Dosimetry

The mean absorbed dose to the kidneys was calculated using the Medical Internal Radiation Dose pamphlet 21 formalism (5) based on our previously published kinetic data of indium 111-octreotide (1) and Monte Carlo simulated S-factors using the MOBY mouse phantom (16).

### Statistical analysis

Results from image analysis of the area of γH2AX-labeled foci, glomerulus count, real-time PCR, RIA, VetScan, and ELISA were evaluated by comparisons of experimental groups using analysis of variance with *post hoc* Tukey's test. For the PCR profiler array, no statistical test was performed due to pooled samples. For histological scoring, the Kruskal–Wallis test with *post hoc* Dunn's test was used. Survival analysis was performed with the Kaplan–Meier estimator, and differences were analyzed using the Mantel–Cox log-rank test.

Statistical comparison was made between control and ^177^Lu-DOTATATE, control and ^177^Lu-DOTATATE+A1M, respectively, and ^177^Lu-DOTATATE and ^177^Lu-DOTATATE+A1M. Only significant differences are presented in the figures. All statistical calculations were performed with GraphPad Prism (GraphPad Prism 7.0; GraphPad Software; GraphPad, Bethesda, MD). Values of *p* < 0.05 were considered significant.

## Supplementary Material

Supplemental data

## Footnotes

^*^ http://imagej.nih.gov/ij/

## Abbreviations Used

γH2AX: H2AX phosphorylated on serine 139
A1M: α_1_-microglobulin
ANOVA: analysis of variance
*Bax*: bcl-2-like protein 4
*Bcl2l1*: Bcl-2-like 1
BUN: blood urea nitrogen
DAPI: 4′,6-diamidino-2-phenylindole
DSB: DNA double-strand break
*Gadd45a*: growth arrest and DNA damage-inducible protein alpha
*GAPDH*: glyceraldehyde 3-phosphate dehydrogenase
*HO-1*: heme oxygenase 1
*Hsp70*: heat shock protein 70
IF: immunofluorescence
IHC: immunohistochemistry
K^+^: potassium ion
^177^Lu: lutetium 177
mRNA: messenger RNA
Na^+^: sodium ion
*NGAL*: neutrophil gelatinase-associated lipocalin
PBS: phosphate-buffered saline
PRRT: peptide receptor radionuclide therapy
qPCR: real-time quantitative PCR
ROS: reactive oxygen species
*TnfRsf10b*: tumor necrosis factor receptor superfamily member 10B
