# Supplementary material for: Protection of Kidney Function with Human Antioxidation Protein α1-Microglobulin in a Mouse 177Lu-DOTATATE Radiation Therapy Model
Source: Antioxid Redox Signal. 2019 Mar 29;30(14):1746–59. doi: 10.1089/ars.2018.7517 (PMC6477591; doi:10.1089/ars.2018.7517)
Supplement: Supplemental data [file Supp_Data.pdf]

Supplementary Data

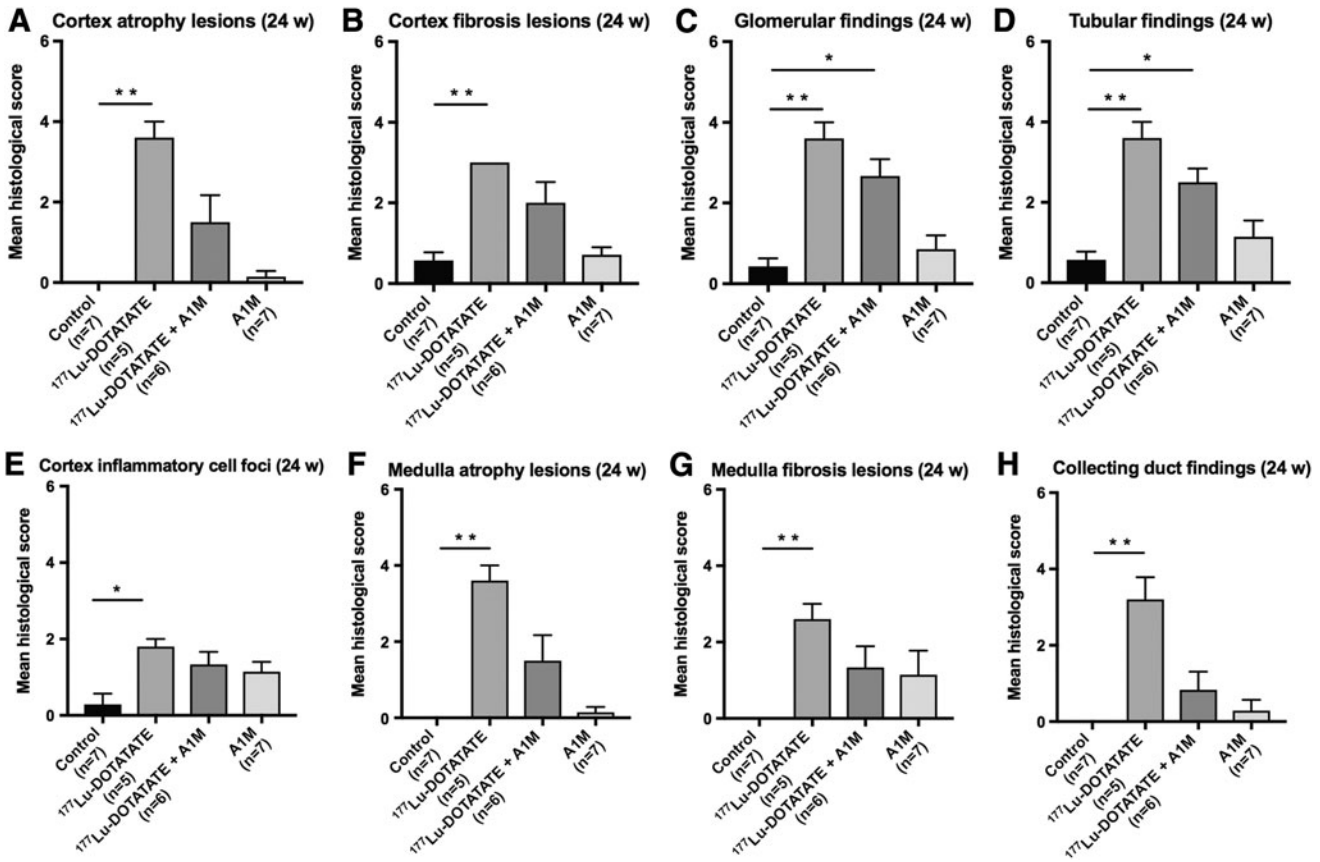

SUPPLEMENTARY FIG. S1. A1M reduces kidney damage after injection with ^{177}Lu-DOTATATE. (**A–H**) Histological scores of eight different kidney lesions found in the four mouse groups. Lesions were separately scored (0–4 severity) and are presented as group mean. Cortex atrophy (**A**), cortex interstitial fibrosis (**B**), glomerular findings (**C**), tubular findings (**D**), cortex inflammatory cell foci (**E**), medulla atrophy (**F**), medullary interstitial fibrosis (**G**), collecting duct findings, and (**H**) scores for animals 24 weeks postinjection. Assessments were performed in hematoxylin–eosin-stained kidney sections (10 μm). Statistical comparison was made between control and ^{177}Lu-DOTATATE, control and ^{177}Lu-DOTATATE+A1M, respectively, and ^{177}Lu-DOTATATE and ^{177}Lu-DOTATATE+A1M. Only significant differences are presented in the figure. Values are presented as mean ± SEM. Differences in groups were analyzed using the Kruskal–Wallis test with *post hoc* Dunn's test for histological scoring (**A–H**). $*p < 0.05$, $**p < 0.01$, A1M, α_1-microglobulin; ^{177}Lu, lutetium 177.

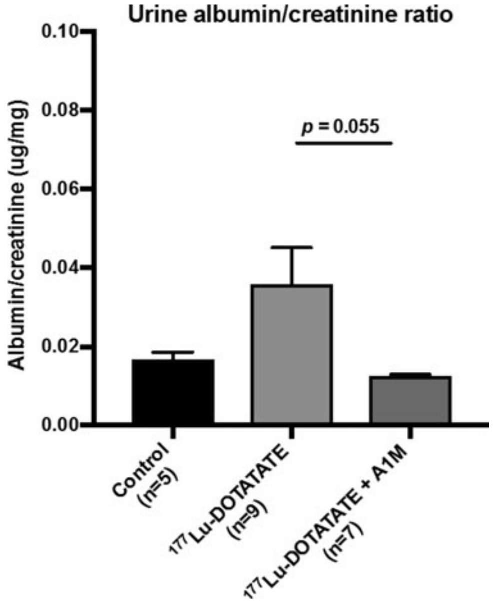

SUPPLEMENTARY FIG. S2. ^{177}Lu-DOTATATE injections in nude BALB/c mice affect urine albumin levels, a marker of proteinuria. Urine level of the functional marker albumin in BALB/c nude mice 4 days postinjections, measured by ELISA and corrected for creatinine levels analyzed using a colorimetric assay. Statistical comparison was made between control and ^{177}Lu-DOTATATE, control and ^{177}Lu-DOTATATE+A1M, respectively, and ^{177}Lu-DOTATATE and ^{177}Lu-DOTATATE+A1M. Differences in groups were analyzed using one-way analysis of variance with *post hoc* Tukey's test.
